# Unsupervised Pre-training of a Deep LSTM-based Stacked Autoencoder for Multivariate Time Series Forecasting Problems

**DOI:** 10.1038/s41598-019-55320-6

**Published:** 2019-12-13

**Authors:** Alaa Sagheer, Mostafa Kotb

**Affiliations:** 1 College of Computer Science and Information Technology, King Faisal University, Al-Ahsa, 31982 Saudi Arabia; 2 Center for Artificial Intelligence and RObotics (CAIRO), Faculty of Science, Aswan University, Aswan, 81528 Egypt

**Keywords:** Electrical and electronic engineering, Computer science

## Abstract

Currently, most real-world time series datasets are multivariate and are rich in dynamical information of the underlying system. Such datasets are attracting much attention; therefore, the need for accurate modelling of such high-dimensional datasets is increasing. Recently, the deep architecture of the recurrent neural network (RNN) and its variant long short-term memory (LSTM) have been proven to be more accurate than traditional statistical methods in modelling time series data. Despite the reported advantages of the deep LSTM model, its performance in modelling multivariate time series (MTS) data has not been satisfactory, particularly when attempting to process highly non-linear and long-interval MTS datasets. The reason is that the supervised learning approach initializes the neurons randomly in such recurrent networks, disabling the neurons that ultimately must properly learn the latent features of the correlated variables included in the MTS dataset. In this paper, we propose a pre-trained LSTM-based stacked autoencoder (LSTM-SAE) approach in an unsupervised learning fashion to replace the random weight initialization strategy adopted in deep LSTM recurrent networks. For evaluation purposes, two different case studies that include real-world datasets are investigated, where the performance of the proposed approach compares favourably with the deep LSTM approach. In addition, the proposed approach outperforms several reference models investigating the same case studies. Overall, the experimental results clearly show that the unsupervised pre-training approach improves the performance of deep LSTM and leads to better and faster convergence than other models.

## Introduction

Recent advances in sensors and measurement technology have led to the collection of high-dimensional datasets from multiple sources recorded over time. Such complex datasets may include several correlated variables; therefore, they are denoted as multivariate time series (MTS) datasets^1^. They are continuously produced in a wide spectrum of industrial, environmental, social, and healthcare applications, such as health monitoring, volatility analysis in financial markets, control systems in automobiles and avionics, and monitoring in data centres, to mention just a few. In such complex applications, one of the key requirements is to maintain the integrity of the sensory data so that it can be monitored and analysed in a trusted manner. In related research, sensor integrity has been analysed by non-parametric methods, such as Bayesian methods^2^. However, it is demonstrated that regression models based on non-parametric methods are highly sensitive to the model parameters^3^. Thus, exploring the optimum parameters of a regression model with multivariate sequential data is still in demand.

The artificial neural network (ANN) is a widely used model employed for the time series forecasting problem in the context of its universal approximation capabilities^4^. conventional ANNs that have shallow architectures are difficult to train if they become too complex, e.g., when the network includes many layers and, consequently, many parameters. In contrast to shallow ANN architectures, it is widely demonstrated that the deep ANN architecture, which is called a deep neural network (DNN), outperforms the conventional shallow ANN architecture in several applications^5^. Recently, deep learning has gained substantial popularity in the machine learning community since it is considered a general framework that facilitates the training of deep neural networks with many hidden layers^6^. The most important advantage of deep learning is that it does not need any hand-crafted features to learn and can easily learn a hierarchical feature representation from the raw data directly^7^.

There are many neural network models that are widely used to solve several types of time series forecasting problems. Among these models, recurrent neural networks (RNNs) have received much attention^4,5^. The reason for such attention is that RNNs are a class of ANN models that possess an internal state or short-term memory due to the recurrent feedback connections, which makes RNNs suitable for modelling sequential or time series data. In such modelling, the RNN maintains a vector of activation parameters for each time step, especially when short-term dependencies are included in the input data. However, if they are trained with stochastic gradient descent, they will have difficulty in learning long-term dependencies that are encoded in the input sequences due to the vanishing gradient problem^8,9^. This problem has been addressed by using a specialized neuron or cell structure in the long short-term memory (LSTM) network that maintains constant backward flow in the error signal that enables the LSTM to learn long-term dependencies^10,11^.

Despite the advantages cited for the LSTM, its performance for time series problems is not always satisfactory^12^. Similar to the shallow RNN, the shallow LSTM architecture cannot represent the complex features of sequential data efficiently, particularly if they are utilized to learn long-interval time series data with high non-linearity. As a recent remedy, the authors of this paper recently presented a deep LSTM model, called DLSTM^5^, capable of circumventing the limitations of the conventional shallow LSTM model. DLSTM includes stacked LSTM layers, one above another, such that each layer contains multiple cells.

Here, it is worth mentioning that our DLSTM model is different from the hierarchical connectionist temporal classification (HCTC) model that was presented a decade ago as the first attempt to stack LSTM layers^13^. HCTC consists of two levels of connectionist temporal classification (CTC) network, where each CTC network uses the bi-directional LSTM and every network has its own softmax layer. To learn the model, every network needs a label, makes its own prediction, and computes an error signal. The model runs when the first CTC network takes as the input the external signal, and every network forwards its predicted label to the next network. In contrast, our DLSTM model^5^ consists of stacked layers (or blocks) of LSTM and only one output layer comprises the final layer. Thus, only the output layer takes a label and makes a prediction. The DLSTM runs when the first LSTM layer takes the input sequence, and every LSTM layer feeds its hidden state to the next LSTM in the stack. The second difference between both models concerns the calculation of the error signal propagated through the stack. The DLSTM calculates only one error signal at the final output layer, and then backpropagates it through all previous layers. However, in the HCTC model, every network in the hierarchy computes an error signal, and then backpropagates it to every lower level. Thus, the final error signal calculated at each network is the sum of its own error signal and the backpropagated signal from the upper networks. The third difference between both models is the design purpose, where HCTC is designed for assigning a sequence of labels to unsegmented data, such as speech or hand-writing^14^, whereas DLSTM is designed for analysing time series data for forecasting or classification applications.

Empirically, the DLSTM model showed better performance compared with RNN, LSTM, deep RNN, and other statistical and ANN modules in performing forecasting, but using univariate time series (UTS) data^5^. Unlike UTS problems, when DLSTM is used to perform forecasting using MTS data, we found that its performance was not satisfactory to some extent. For this reason, using LSTM, either shallow or deep architectures, in modelling MTS problems is not common in the literature. The reason for this is due to the way that the neurons in the DLSTM model are initialized. It is widely demonstrated that the neurons’ weights in most standard ANN approaches are initialized randomly in the context of learning using stochastic gradient descent^15^. In such cases, the backpropagation algorithm may be trapped within multiple local minima, particularly when several non-linear variables are modelled^16^. Accordingly, sincere efforts are exerted to investigate new methods for weight initialization in standard ANN approaches^15–17^.

Therefore, there is little doubt that DLSTM performs well in UTS classification problems^5^ where only one variable or feature is modelled. In contrast, the features in MTS classification problems, with several variables, are highly correlated. The random initialization of a large numbers of neurons in such situations will lead the learning algorithm to converge to different local minima, depending on the values of the parameter initialization. Furthermore, and as a general practice, previous studies have demonstrated that training deep networks with several layers using random weights initialization and supervised training provide worse results than training shallow architectures^8,18,19^.

Several treatments have been introduced in order to facilitate either the architecture or the training of deep learning models that suffer from all the aforementioned limitations. One of the earlier treatments that facilitated supervised learning by unsupervised pre-training of a hierarchical RNN architecture is known as the chunker-automatizer module^20^. This module uses a gradient descent optimizer to compress the learned behaviour of the chunker RNN layer into the automatizer RNN layer. This procedure learns to imitate the chunker network and repeats training to avoid forgetting of previous skills. At that early time, this learning fashion was known as collapsing (or compressing) the behaviour of one learner into another^20^, similar to the recent learning approach of the teacher-student learner, known as knowledge distillation^21^. Certainly, this enables data compression and, thereby, the vanishing gradient problem of RNN becomes less relevant.

More than a decade later, another approach applied an unsupervised greedy layer-wise pre-training algorithm to the deep belief network (DBN)^22,23^. The proposed DBN shows a successful performance in computer vision applications. The rectified linear units^24^ and the Maxout networks^25^ are two other treatments, as well. When they are applied to the convolutional neural network (CNN), they were shown to be useful to reduce the impact of vanishing gradient in deep models. Stacking many convolutional layers^26^ instead of fully connected layers is an effective treatment to overcome this problem in deep neural networks^19^.

Motivated by the success of unsupervised pre-training in data compression^20^, dimensionality reduction^20,27^, classification^20,28^, and UTS forecasting^20^ problems, we extend it to the regression problem via deep recurrent neural networks solving the MTS forecasting problem. In this paper, we introduce our previous model DLSTM in an unsupervised pre-training fashion based on a stacked autoencoder training architecture to avoid the random initialization of LSTMs’ units. We tested the proposed model to solve the MTS forecasting problem using two real-world datasets. We conduct a comparison between the proposed approach and our previous model DLSTM, where the impact of replacing the randomized supervised method in DLSTM with the unsupervised pre-training method is promising. Furthermore, we conduct a comparison with other reported regression baselines and reference models that solve the same problems using the same datasets, indicating a clear superiority of the proposed approach using three different performance metrics. The main contributions of this paper are as follows:

1. Develop an unsupervised pre-training framework for our previous model DLSTM^5^.
2. Present a new LSTM-based autoencoder learning approach to solve the random weight initialization problem of DLSTM.
3. Propose a robust forecasting module based on unlabelled time series data, which could be used to convert the time series observations into representative features that can be utilized easily for further use and analysis.

The rest of the paper is organized as follows: Section 2 briefly summarizes the related work to this paper. The MTS problem and the proposed methodology are presented in section 3. Section 4 shows the experimental settings and datasets used in this paper. The experimental results of two case studies are provided in section 5. Discussion and analysis of the results are given in section 6 and, finally, the paper is concluded in section 7.

## Related Work

It is demonstrated that analysing UTS data is easy and common; however, analysing MTS data is complex due to the correlated signals involved^29^. The key challenge in MTS problems is to model such complex real-world data, as well as to learn the latent features automatically from the input correlated data^3^. For this reason, the machine learning community has afforded scant attention to the MTS forecasting problem versus much attention to forecasting problems using UTS data. This section shows an overview of state-of-the-art methods that adopt the unsupervised pre-training procedure to facilitate the overall learning process. Then, the section shows the recent achievements of state-of-the-art techniques that are implemented to solve the MTS forecasting application using same datasets utilized in this paper to assess the proposed model.

### State-of-the-art models

It is widely known that the conventional ANN does not provide a very good result when the function to be approximated is very complex, especially in regression problems^30^. There are various reasons for this, such as the neurons’ initialization or the overfitting or the function becoming stuck in local minima^15^. As such, different training approaches along with different network architectures for the conventional ANNs are introduced^4,31,32^. One of these approaches, as a trial towards a fine analysis for complex real-world data, is to develop robust features that are capable of capturing the relevant information from data. However, developing such domain-specific features for each task is expensive, time-consuming, and requires expertise in working with the data^12^. The alternative approach is to use an unsupervised feature learning strategy to learn the feature representation layers from unlabelled data, which was early presented by Schmidhuber^14,20^. In fact, the latter approach has many advantages, such as conducting learning based on unlabeled data, and having the availability and abundance of unlabelled data, in contrast to labelled data. In addition, learning the features from unlabelled data in advance, which is called pre-training, is much better than learning them from hand-crafted features^33,34^.

Beside these advantages, the most important advantage is the ability to stack several feature representations layers to create deep architectures^20^, which are more capable of modelling complex structures and correlated features that are included in MTS problems^12,30^. These unsupervised pre-training approaches alleviate the underfitting and overfitting problems that had restrained the modelling of complex neural systems for a period of time^35^. Most of the current unsupervised pre-training models are developed in a layer-wise fashion, which is enough to train simple models, and then stack them layer-by-layer to form a deep structure.

Hinton *et al*. developed a greedy layer-wise unsupervised learning algorithm for deep belief networks (DBNs), a generative model with many layers of hidden variables^22^. Hinton’s approach consists of a stack of restricted boltzmann machines (RBMs), where two consecutive learning steps are conducted: a pre-training step, which is a kind of unsupervised learning using the gradient of the network energy of the RBM, and a fine-tuning step, which is a kind of supervised learning step to calculate the backpropagation errors. Then, the learned feature activations of an RBM layer are used as the input layer to train the following RBM in the stack^22^.

Next, Bengio *et al*. presented a theoretical and experimental analysis for Hinton’s approach, asserting that the greedy layer-wise pre-training approach will help to optimize the modelling of the deep networks^23^. In addition, Bengio concluded that this approach can yield a good generalization because it initializes the upper layers with better representations of relevant high level abstractions. Moreover, Ehran *et al*. tried specifically to answer the following question: how does unsupervised pre-training work? They empirically showed the effectiveness of the pre-training step and how it guides the learning model towards basins of attraction of minima that support a good generalization from the training dataset^33^. Furthermore, they showed evidence that the layer-wise pre-training procedure performs the function of regularization perfectly. Sarikaya *et al*. applied Hinton’s DBN model to the natural language understanding problem and compared it with three text classification algorithms, namely, support vector machines (SVM), boosting, and maximum entropy^36^. Using the additional unlabelled data for DBN pre-training and combining DBN-based learned features with the original features provides significant gains to DBN over the other models.

Le Paine *et al*. empirically investigated the impact of unsupervised pre-training in contrast to a number of recent advances, such as rectified linear units (ReLUs), data augmentation, dropout, and large labelled datasets^34^. Using CNNs, they tried to answer the question: When is unsupervised pre-training useful given recent advances? Their investigation, in an image recognition application, was based on three axes: first developing an unsupervised method that incorporates ReLUs and recent unsupervised regularization techniques. Second, analysing the benefits of unsupervised pre-training compared to data augmentation and dropout on a benchmark dataset, while varying the ratio of unsupervised to supervised samples. Third, verifying their findings using benchmark datasets. They found that unsupervised pre-training is a perfect way to improve the performance, especially when the ratio of unsupervised to supervised samples is high^34^. Tang *et al*. presented a new pre-training approach based on knowledge transfer learning^35^. In contrast to the layer-wise approach, which trains the model components layer-by-layer, their approach trains the entire model as a whole. In their experiments on speech recognition, they could learn complex RNNs as a student model by a weaker deep neural network (DNN) as a teacher model. Furthermore, their approach can be combined with a layer-wise pre-training of CNN to deliver additional gains.

Recently, Saikia *et al*. empirically analysed the effect of different unsupervised pre-training approaches in modelling regression problems using four different datasets^30^. They considered two methods separately, namely, DBN and stacked autoencoder (SA), and compared their performance with a standard ANN algorithm without pre-training. Liu *et al*. applied Hinton’s model on stacked autoencoders (SAEs) to solve gearbox fault diagnosis^37^. The proposed method can directly extract salient features from frequency-domain signals and eliminate the exhaustive use of hand-crafted features. Meng *et al*. used it also in order to train the stacked denoising sparse autoencoder layer-by-layer^38^.

As we mentioned in this section, most of the previous work in unsupervised pre-training NN (or deep NNs) has focused on data compression^20^, dimensionality reduction^20,27^, classification^20,28^, and UTS forecasting^20^ problems. Importantly, time series forecasting with deep learning techniques is an interesting research area that needs to be studied as well^19,26^. Moreover, even the recent time series forecasting research in the literature has focused on UTS problems. Very few works in the literature are devoted to forecasting via MTS data. For example, Romeu *et al*. used a pre-trained autoencoder-based DNN for UTS forecasting of indoor temperature^26^. Kuremoto *et al*. used the earlier Hinton model for unsupervised pre-training step and backpropagation for the fine-tuning step using an artificial UTS dataset^39^. Malhotra *et al*. extended an approach that uses a CNN with data from audio and image domains to be used in the time series domain and trained a multilayer RNN that can then be used as a pre-trained model to obtain representations for time series^40^. Their method overcomes the need for large amounts of training data for the problem domain. Wang *et al*. used Hinton’s DBN model in a cyclic architecture instead of the single DBN model that was earlier presented by Hinton^41^. However, while they used MTS data in their experiments, their target was a classification problem and not a forecasting problem, such as our target in this paper. In addition, their model architecture is completely different from ours as presented here.

### Overview of the reference models

Furthermore, given the complexity of MTS data that are described in the previous section, few works have been introduced to solve forecasting applications using MTS datasets. Indeed, specific methodologies are required for treatment rather than traditional parametric methods, auto-regressive methods, and Gaussian models^2,42^. In the following, we briefly review previous contributions in the literature that are used to solve the same forecasting problems that are studied in this paper.

Wu *et al*. presented a hybrid prediction model that combines a natural computation algorithm, namely, the artificial immune system (AIS) with regression trees (RT)^43^. The cells in AIS represent the basic constituent elements of the model and the RT forecasting sub-models are embedded in the AIS model to form the cells’ pool. They applied their hybrid model to solve the bike sharing system problem, which we also solve in this paper. Huang *et al*. developed a deep neural network model that integrates the CNN and LSTM architectures to solve the PM2.5 mass concentrations forecasting problem^44^. In this simple combination, the CNN is used for feature extraction and the LSTM is used to analyse the features extracted by the CNN, and then predict the PM2.5 concentration of the next observation. The authors added a batch normalization step to improve the forecasting accuracy. The proposed hybrid approach outperformed the single CNN and LSTM models.

Qi *et al*. proposed a hybrid model that integrates graph convolutional networks (GCN) and long short-term memory (LSTM) networks to model and forecast the spatio-temporal variation of the PM2.5 mass concentrations forecasting problem^45^. The role of the GCN is to extract the spatial dependency between different stations, whereas the role of the LSTM is to capture the temporal dependency among observations at different times. In a subsequent section of our paper, we also treat the PM2.5 concentrations problem. Cheng *et al*. developed three hybrid models combine statistical learning approaches with machine learning approaches to forecast the PM2.5 mass concentrations problem^46^. These models are: wavelet-ANN, wavelet-ARIMA, and wavelet-SVM. Their experimental results showed that the proposed hybrid models can significantly improve the prediction accuracy better than their single counterpart models. In addition, the wavelet-ARIMA model has the highest accuracy compared to the other two hybrid models.

Last, Zhao *et al*. proposed a hybrid method that combines five modules to solve the PM2.5 mass concentrations forecasting problem^47^. These five modules are the data preprocessing module, feature selection module, optimization module, forecasting module and evaluation module. They used the complete ensemble empirical mode decomposition with adaptive noise and variational mode decomposition (CEEMDAN-VMD) to decompose, reconstruct, identify and select the main features of PM2.5 observations through the data preprocessing module. Then, they used the auto-correlation function (ACF) to extract the variables that have relatively large correlation with the predictor, thereby selecting the input variables according to the order of correlation coefficients. Next, the least squares support vector machine (LSSVM) is used to predict the future points of concentrations. Finally, the parameters of the LSSVM are optimized using the whale optimization algorithm (WOA).

It is clear that most of the aforementioned state-of-the-art methods in the literature that are used to solve the same MTS forecasting problems are hybrid methods that combine more than a model, in contrast to the model proposed in this paper, which is a standalone model. In summary, undoubtedly, the layer-wise architecture that was earlier presented by Hinton has a concrete theoretical foundation as well as an empirical assessment. However, it is not easy to employ such a layer-wise structure to pre-train models without a clear multilayer structure^35^. In the following section, we propose a novel layer-wise structure for the LSTM-based autoencoder in a deep architecture fashion. We will show that the proposed approach outperforms our previous deep LSTM model as well as other reported baseline approaches.

## Problem and Methodology

### The multivariate time series (MTS) forecasting problem

Time series data comprise a sequence of observations recorded in uniform intervals over a period of time. A time series forecasting problem is the task of predicting future values of time series data either using previous data of the same signal (UTS forecasting) or using previous data of several correlated signals (MTS forecasting)^29^. Mathematically, the UTS problem can be formalized as a sequence of *n* real-valued numbers $x=\{x(i)\in  {\mathcal R} :i=1,2,\mathrm{..}.,n\}$ and *n* represents the length of the series that is recorded for the corresponding pattern. In UTS forecasting problems, the forecast model usually predicts the variable of interest *x* using past values that precede *x*. In this manner, the forecasting model perceives the structure and learns the trend of the underlying pattern and extrapolates the interested process into the future.

The MTS forecasting problem is very common in real life applications. Intuitively, it is more complex since it has two or more variables. It is defined as a finite sequence of UTS problems, such that each UTS problem represents a pattern. In a formal way,

1 $$X=({x}_{1},{x}_{2},\ldots ,{x}_{m})$$

represents an MTS that includes *m* variables, where the corresponding component of the $jth$ variable ${x}_{j}$ is a UTS problem of length *n* and can be given as:

2 $$x=\{{x}_{j}(i)\in  {\mathcal R} :i=\mathrm{1,}\,2,\ldots ,n\}(j=\mathrm{1,}\,\mathrm{2,}\ldots ,m\mathrm{)}.$$

In MTS forecasting problems, as in this paper, the forecast model will predict the interested variable *x*_*k*_ not only using its past values but also using values of other variables $({x}_{1},{x}_{2},\,\mathrm{...,}\,{x}_{m})$^19^.

### The long short-term memory (LSTM)

LSTM is the elegant variation of the RNN architecture^11^, which is a recursive neural network approach that can be applied for the modelling of sequential data. The key feature of an RNN is the network delay recursion, which enables it to describe the dynamic performance of systems^48^. In addition, RNNs maintain a vector of activations for each time step, which makes the RNN an extremely deep neural network^49^. However, it is often difficult to train RNNs to learn the long-term dependencies in time series data due to the exploding and the vanishing gradient problems^8,9,50^. Both problems are caused by the iterative property of the RNN, whose gradient is essentially equal to the recurrent weight matrix but raised to a high power. These matrix powers cause the gradient to grow or to shrink at a rate that is proportional to the number of time-steps in the dataset^49^.

Nevertheless, it is demonstrated that the exploding gradients problem is relatively simple to cope with using a technique known as gradient clipping, which simply shrinks the gradients whose norms exceed a threshold^50^. One of the advantages of the gradient clipping technique is keeping the gradient small to some extent for most of the learning time, whereas the learning or convergence will suffer if the gradient is reduced too much^49^. In contrast, the problem of the vanishing gradient is extremely difficult because it causes the gradient’s component in the trends that correspond to long-term dependencies to be minor, whereas it causes the gradientâ€™s component in the trends that correspond to short-term dependencies to be large. For this reason, the RNN can learn the short-term dependencies easily, but it suffers with long-term dependencies^8,9,51^.

**Figure 1:**
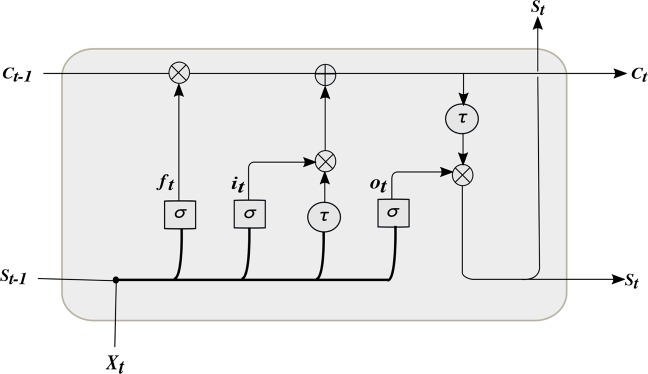
The LSTM block, where ${f}_{t},{i}_{t},{o}_{t}$ are forget, input, and output gates respectively.

The design of the LSTM makes it an effective solution to combat the vanishing gradient problem of the RNN^8,9^. It uses a memory cell capable of representing the long-term dependencies in sequential data. As depicted in Fig. 1, the LSTM memory cell is composed of four gates (or units); namely, the input gate, the output gate, the forget gate, and the self-recurrent neuron. These gates are responsible for controlling the interactions among different memory units. Specifically, the input gate controls whether the input signal can modify the state of the memory cell or not. In contrast, the output gate controls whether it can modify the state of other memory cells or not. Whilst the forget gate can choose to forget (or remember) its previous status. The gates, hidden outputs, and cell states can be represented as follows^11^:

3 $${f}_{t}=\sigma ({X}_{t}{U}^{f}+{S}_{t-1}{W}^{f}+{b}_{f})$$

4 $${i}_{t}=\sigma ({X}_{t}{U}^{i}+{S}_{t-1}{W}^{i}+{b}_{i})$$

5 $${o}_{t}=\sigma ({X}_{t}{U}^{o}+{S}_{t-1}{W}^{o}+{b}_{o})$$

6 $${\tilde{C}}_{t}=\,\tanh \,({X}_{t}{U}^{c}+{S}_{t-1}{W}^{c}+{b}_{c})$$

7 $${C}_{t}={C}_{t-1}\otimes {f}_{t}\oplus {i}_{t}\otimes {\tilde{C}}_{t}$$

8 $${S}_{t}={o}_{t}\otimes \,\tanh \,({C}_{t})$$

where $({U}^{f},{U}^{i},{U}^{o},{U}^{c})$, $({W}^{f},{W}^{i},{W}^{o},{W}^{c})$ and $({b}_{f},{b}_{i},{b}_{o},{b}_{c})$ are input weights, recurrent weights and biases, respectively. *X*_*t*_, *S*_*t*_ and *C*_*t*_ are input, hidden and cell state at time step *t*, respectively. ${S}_{t-1}$ and ${C}_{t-1}$ are the hidden and cell state at time step $t-1$, respectively. $\otimes$, $\oplus$ and $\sigma$ are pointwise multiplication, pointwise addition and sigmoid activation, respectively.

It is demonstrated that there are other attempts to overcome the vanishing gradient problem of RNN; however, the LSTM is the most efficient attempt. The LSTM solves this problem by truncating the gradients in the network where it is innocuous to do so by enforcing the constant error flows through the constant error carousels within special multiplicative units. These special non-linear units learn whether to open or close the gates of the network in order to adjust such a constant error flow. Briefly, we can say that the LSTM approximates the long-term information with a significant delay by speeding up the conventional RNN algorithm^52^.

### The autoencoder (AE)

**Figure 2:**
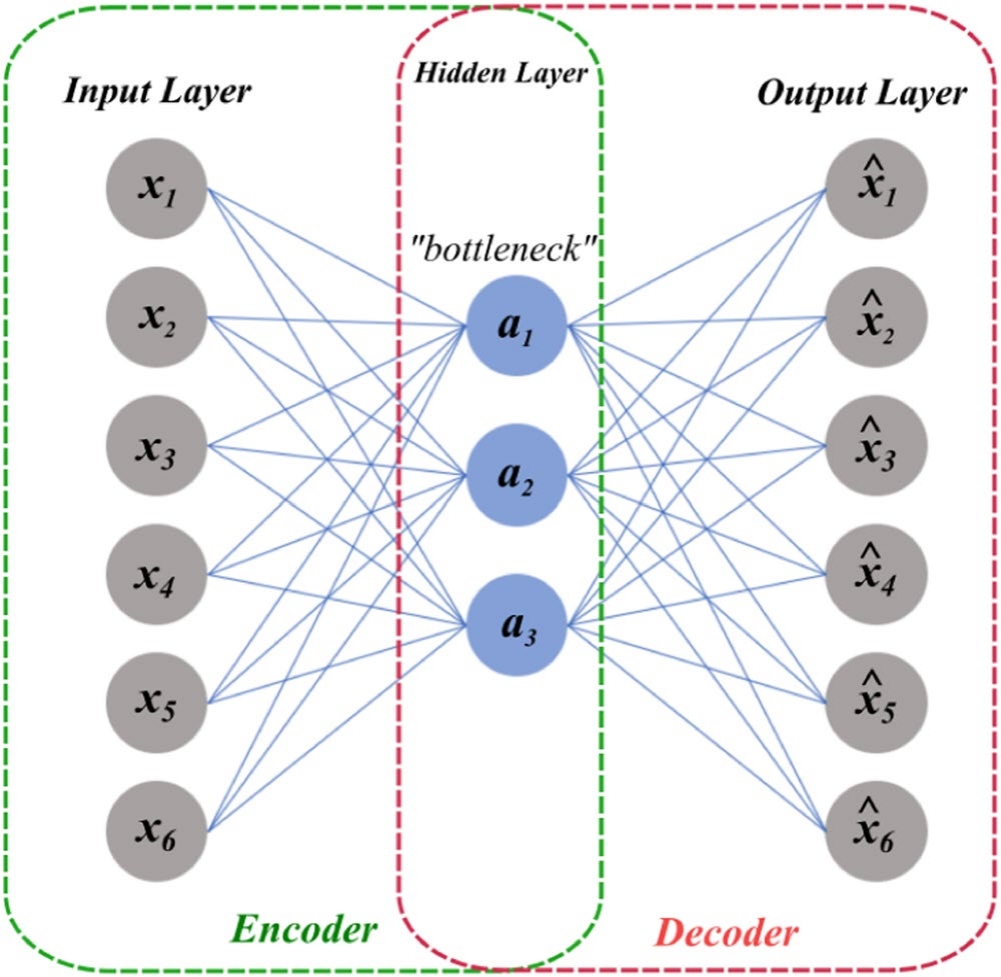
The autoencoder architecture.

The AE is an ANN that consists of a sequentially connected three-layer arrangement: the input layer, the hidden layer, and the output (or reconstruction) layer, where all layers are working in an unsupervised learning paradigm, as shown in Fig. 2. The AE is often used for data generation, or as a generative model, where its training procedure consists of two phases: encoding, in which the input data are mapped into the hidden layer, and decoding, in which the input data are reconstructed from the hidden layer representation. In the encoding phase, the model learns a compressed representation (or latent variables) of the input. In contrast, in the decoding phase, the model reconstructs the target from the compressed representation during the encoding phase.

Given an unlabelled input dataset *X*_*n*_, where *n* = 1, 2, …, *N* and ${x}_{n}\in { {\mathcal R} }^{m}$, the two phases can be formulated as follows:

9 $$h(x)=f({W}_{1}x+{b}_{1})$$

10 $$\hat{x}=g({W}_{2}h(x)+{b}_{2})$$

where $h(x)$ represents the hidden encoder vector calculated from input vector *x*, and $\hat{x}$ is the decoder (or reconstruction) vector of the output layer. Additionally, *f* is the encoding function and *g* is the decoding function, ${W}_{1}$ and ${W}_{2}$ are the weight matrix of the encoder and decoder, respectively, and ${b}_{1}$ and ${b}_{2}$ are the bias vectors in each phase, respectively^37^.

The difference between the input and the reconstructed input (or output) is usually called the reconstruction error. This reconstruction error is represented in the form of an objective function, where during training, the model tries to reduce it, e.g. to minimize $||x-\hat{x}{||}^{2}$. Stacking multiple AE layers is possible such that useful high level features are obtained, with some qualities such as abstraction and invariance. In such a case, a lower error reconstruction will be obtained, and thereby, better generalization is expected^53^.

### LSTM-based stacked autoencoder (LSTM-SAE)

This section shows the proposed architecture and learning algorithm of the proposed model LSTM-SAE.

#### Architecture

**Figure 3:**
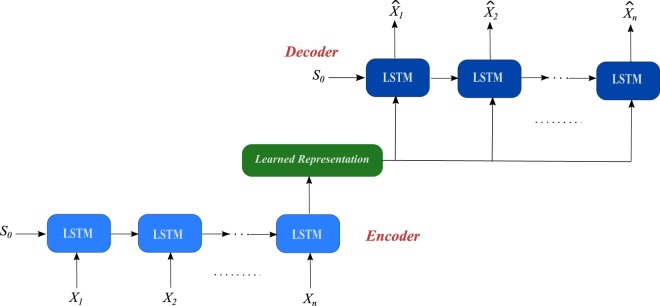
LSTM-based autoencoder architecture.

Here, we propose a new architecture as a variation of the original architecture of the autoencoder to enable it to extract features from MTS, or generally, time series problems. In particular, we changed the original AE architecture from the feed forward neural network base, as depicted in Fig. 2, into the LSTM recurrent network base, as depicted in Fig. 3, and we denote it as the LSTM-based autoencoder (LSTM-AE). LSTM-AE relies on the concept that the recurrent network is more suitable for modelling time series data. As shown in Fig. 3, the LSTM-AE architecture consists of two LSTM layers; one runs as an encoder and the other runs as a decoder. The encoder layer takes the input sequence (${x}_{1},{x}_{2},\,\mathrm{...,}\,{x}_{n}$) and encodes it into a learned representation vector. Then, the decoder layer takes that vector as input and tries to reconstruct the input sequence into (${\hat{x}}_{1},{\hat{x}}_{2},\,\mathrm{...,}\,{\hat{x}}_{n}$). The cost function of the LSTM-AE is the mean squared error of the difference between the original input sequence and the reconstructed sequence. Motivated by the first pre-training hierarchical RNN approach early presented by Schmidhuber^14,20^ as well as the greedy layer-wise unsupervised pre-training DBN approach presented by Hinton *et al*.^22^, we are stack more than an LSTM-AE layer in a deep fashion and call it as LSTM-based stacked autoencoder (LSTM-SAE).

It is worth mentioning that the proposed LSTM-AE is different from the sequence-to-sequence model that was presented recently by Sutskever *et al*.^54^. Indeed, both models use two LSTM layers: one LSTM as an encoder and the other LSTM as a decoder in a seq-to-seq fashion. Sutskever’s model is designed essentially for language modelling, so that the decoder layer is an LSTM-based language model (LSTM-LM), and it computes the conditional probability of the outputs given the input sequence from the learned representation vector by applying a softmax output layer. In contrast, the decoder layer in the proposed LSTM-AE reconstructs the input sequence from the learned representation without using any softmax or output layers. Another difference is that, the initial hidden state of the decoder in the LSTM-LM is set to the learned representation (see Fig. 1 in Sutskever’s paper^54^), whereas the initial hidden state in our LSTM-AE model is randomly initialized(${S}_{0}$ in Fig. 3). The most important difference is related to the architecture of both networks, where the seq-to-seq model^54^ uses a multilayered LSTM, namely, four layers for both the encoder and decoder layers. In contrast, the proposed LSTM-AE model uses a shallow LSTM, namely, one LSTM layer for the encoder and decoder layers. Certainly, this distinguishes the proposed LSTM-AE from a computational complexity point of view.

#### Learning

The learning algorithm of the LSTM-SAE model consists of two phases: a pre-training phase and a fine-tuning phase.

- ***Greedy Layer-wise Pre-training Phase:***

In the pre-training phase, we construct a greedy layer-wise structure to train three LSTM-SAE blocks, as shown inFig. 4. The pre-training procedure can be summarized in the following four steps:

1. Train the first LSTM-AE block in the stack; then, save its LSTM encoder layer to be used as input for the second LSTM-AE block in the stack.
2. Load the saved encoder layer and use it to encode the inputs; then, train the second LSTM-AE block in the stack with the encoded version of inputs, and train it to reconstruct the original inputs, not the encoded inputs, in order to enforce the encoder to learn the features of the original inputs. Then, save its LSTM encoder layer to use it as input for the third LSTM-AE block in the stack.
3. Load the two saved encoder layers and use them to encode the inputs twice; then, train the third LSTM-AE block with the encoded version of inputs to reconstruct the original inputs, and save its LSTM encoder layer.
4. Use the three saved LSTM encoders to initialize a three-layer DLSTM model^5^; in the same way described in the training phase of that model.

**Figure 4:**
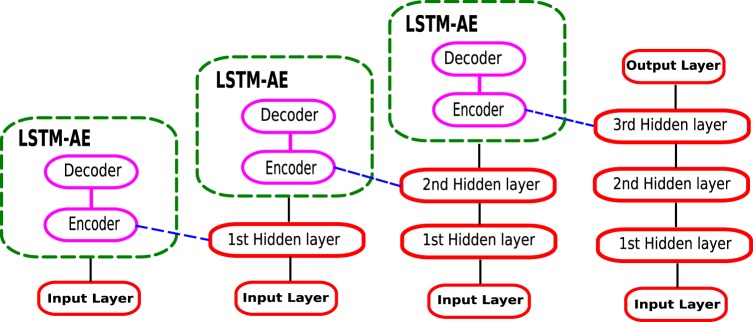
The greedy layer-wise pre-training of LSTM-SAE model.

Obviously, this phase can be generalized for more than three layers, if needed.

- ***Fine-tuning Phase:***

At the end of the pre-training phase, we manage to initialize three hidden layers the DLSTM model. The fine-tuning phase starts by adding an output layer on top of the three hidden layers of the DLSTM model, where the output layer consists of just one neuron, as long as the problem at hand is a regression problem. Then, we start to fine-tune the model in a supervised learning fashion, where the input is a sequence of MTS data in time step *t* and the label is the value of the corresponding variable (*the variable that we need to predict its future values*) from the MTS data in the next time step *t* + 1. In this phase, we evaluate the prediction of DLSTM using out-of-sample testing data. The results that are recorded and compared in the experiments section in this paper are the results of this phase using the testing data.

**Figure 5:**
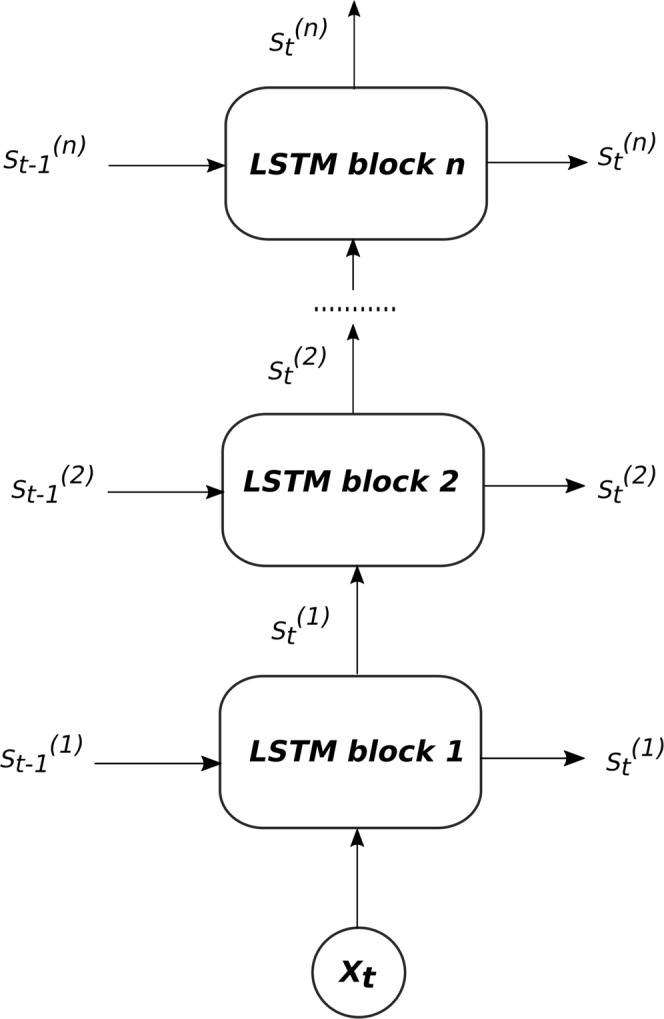
The architecture of DLSTM recurrent network^5^.

It is worth noting here that the proposed model LSTM-SAE is an extension to our previously presented model DLSTM^5^ that is shown in Fig. 5. The difference here is that the LSTM-SAE uses unsupervised pre-trained LSTMs in an autoencoder fashion, rather than the random initialization fashion that was adopted in the DLSTM model. In the experiments section of this paper, we will compare the performance of the original DLSTM^5^, without the pre-training phase, with the pre-trained DLSTM proposed in this paper to precisely ascertain how the pre-training phase enhances the overall DLSTM’s performance.

It is worth mention that, there are some differences between the proposed LSTM-SAE model and the hierarchical RNN model that early presented by Schmidhuber^20^, particularly in the unsupervised pre-training phase. The first difference concerns the objective of the prediction task of Schmidhuber’s model, which is predicting the next input from the previous inputs. In contrast, the LSTM-SAE model tries to reconstruct the inputs by establishing the LSTM autoencoder. Nevertheless, the major difference lies in the way of pre-training scenario; Schmidhuber’s model starts when the first (lowest-level) RNN takes the external input and tries to predict the next input. The second (higher-level) RNN can’t be established unless the first RNN stops improving its predictions. At a given time step, if the first RNN fails to predict the next input, the updating of the first RNN’s weights is stopped. Then, the second RNN takes, as input, the concatenation of this next input plus a unique representation of the corresponding time step. Following this procedure, higher levels are adding to the hierarchy. In contrast, the LSTM-SAE model starts by training the first LSTM-AE to reconstruct the original inputs, then training the second LSTM-AE by the encoded version of the inputs to reconstruct the original inputs, and so on, in the same way as described in the pre-training phase.

## Experimental Settings

This section shows the experimental settings, the datasets, and the parameters selection of the proposed model. In addition, the section gives a brief overview for the performance metrics that will be used in the validation and comparisons in this paper.

### Datasets

For validation purposes, two different case studies, where each case study uses a benchmark dataset, are used to validate the proposed and reference models. For the step of data preprocessing, we adopted the same data preprocessing scheme that we described in our previous paper^5^. The two datasets are as follows.

#### The capital bike sharing dataset

This dataset is a public dataset available in UCI Machine Learning Repository^55^ provided by Fanaee-T *et al*.^56^. They provide a two-year usage log of a bike sharing system, called capital bike sharing (CBS) in Washington D.C., USA. They calculated the number of rental bikes, weather states, and the hourly calendar during the time period between Jan 1st, 2011 to Dec 31st, 2012^56^. It is a multivariate dataset that consists of ten variables, as follows: *Season, Holiday, Weekday, Working day, Weather sit, Temperature in Celsius, Feeling temperature in Celsius, Humidity, Wind speed, and Count of total rental bikes*. The total number of data samples is 17,379 observations, which we split into three sets of 10,512 for training, 2628 for validation, and 4238 for testing. In the experiments with this dataset, the requirement is to predict the future hourly total number of rented bikes given the scope of the other variables.

#### The PM2.5 concentration in the air of CHINA dataset

This dataset is another benchmark public dataset that is widely used in many disciplines in the literature. Its importance stems from its inclusion of the hourly concentration observations in the air of several cities in China; therefore, it is a massive dataset. In this paper, we will consider only the data samples of the city of Beijing, where the observations were collected in the time period between Jan 1st, 2010 to Dec 31st, 2014^57^. This dataset is a multivariate dataset that consists of eight variables, as follows: *PM2.5 concentration, Dew Point, Temperature, Pressure, Combined Wind Direction, Cumulated Wind Speed, Cumulated hours of snow, and Cumulated hours of rain*, and we integer-encoded Wind Direction. The dataset includes a total number of 43,800 observations, which we split into sets of 30,660 for training, 4380 for validation, and 8760 for testing. In the experiments with this dataset, the requirement is to predict the future hourly concentration given the scope of the observations of the other variables.

### Performance measures

In the time series forecasting problem, two kinds of errors are usually measured, in order to estimate the forecasting precision and performance evaluation of the forecasts, namely, scale-dependent error and percentage error. Due to their importance for our subject, we will devote this section to describe both of them

1. *The scale-dependent error measure*

The scale-dependent errors are on the same scale as the data themselves. Therefore, as a limitation, the accuracy measures that are based directly on this error cannot be used to make comparisons between series that are on different scales. In this paper, two known scale-dependent error measures have used, namely, the root mean square error *RMSE* and the mean absolute error *MAE*^58^.

***RMSE*** measures the average magnitude of the errors. Specifically, it is the square root of the average of squared differences between theprediction and actual observations. Therefore, the *RMSE* will be more useful when large errors are particularly undesirable. *RMSE* can be represented mathematically as follows

11 $${\bf{RMSE}}=\sqrt{\frac{1}{n}\mathop{\sum }\limits_{i=1}^{n}\,{({y}_{i}^{obs}-{y}_{i}^{pred})}^{2}}$$

where ${y}_{i}^{obs}$ and ${y}_{i}^{pred}$ are the actual and predicted observations, respectively.

***MAE*** measures the average magnitude of the errors in a set of predictions, regardless of their direction. Therefore, it is the average over the test sample of the absolute differences between the prediction and actual observation where all individual differences have equal weight.

12 $${\bf{MAE}}=\frac{\mathop{\sum }\limits_{i=1}^{n}\,|{y}_{i}^{obs}-{y}_{i}^{pred}|}{n}$$

Based on the definition of both measures, we can note that both *RMSE* and *MAE* are negatively oriented scores and they express average prediction errors. However, it is widely demonstrated in the literature that the *MAE* error measure is more preferred than the *RMSE* error measure, for the following reasons^59^:

1. The *MAE* measure is steadier than *RMSE*, whereas the latter increases as the variance that is associated with the frequency distribution of error magnitudes increases.
2. The *RMSE* measure is related to the sample size, where it has a tendency to be increasingly larger than *MAE* as the test sample size increases. This can be a problem when using *RMSE* with different-sized test samples, which is frequently the case in real-world situations.
3. The *RMSE* gives a relatively high weight to large errors (in bad prediction situations), since the errors are squared before they are averaged. This may skew the metric towards overestimating the modelâ€™s badness. This is particularly problematic behaviour if we have noisy data samples. In contrast, *MAE* penalizes huge errors but not as badly as *RMSE* does. Thus, it is not that sensitive to outliers as *RMSE* is, as long as it does not make use of a square.
4. The *MAE* measure is easier to interpret and justify than *RMSE*.
  (2) *The percentage error measure*

The percentage error measures are more accurate and efficient in tracking the forecasting precision and performance evaluation of the forecasts, since they have the advantage of being scale-independent. Therefore, these kinds of error measures are frequently used to make comparison among forecast models, especially when different scaled datasets are used^58^. One of the most common percentage errors is the symmetric mean absolute percentage error (SMAPE)^60^.

13 $${\bf{SMAPE}}=\frac{100}{n}\mathop{\sum }\limits_{i=1}^{n}\,\frac{{y}_{i}^{obs}-{y}_{i}^{pred}}{{y}_{i}^{obs}+{y}_{i}^{pred}}$$

### Parameters selection

During the validation experiments of this paper, there are a few hyper-parameters that need to be selected. Specifically, these parameters are:

1. Sequence length (lag)
2. Batch size
3. Number of units in the encoder layer
4. Number of epochs for training the LSTM-SAE in the pre-training phase
5. Number of epochs for training the pre-trained model in the fine-tuning phase
6. Dropout rate *(we use a dropout layer after each LSTM layer in the fine-tuning phase)*.

For these hyper-parameters selection, we used the *Hyperopt: Distributed Asynchronous Hyper-parameter Optimization* library^61^, along with using the *RMSE* on the validation data as a criterion for selecting the hyper-parameters. For both phases of pre-training and fine-tuning, we used the *ADAM* optimizer^62^ and the mean squared error (MSE) as a cost function. For the implementation phase of our model, we used the *Keras* library^63^.

## Experimental Results

This section shows the empirical results of our experiments that assess the proposed model. In this evaluation phase, we assess the performance of the LSTM-SAE model and compare its performance with the reference model DLSTM^5^ to solve the same problems using the same datasets that are described in the previous section. More comparisons with other baseline and reference models using the same datasets are given in the following section. To provide a fair evaluation and comparison, the aforementioned experimental settings of all experiments are unified, and we utilized the three performance measures described in the previous section. All tables in this section show the results based on the testing (or unseen) data.

### Case study-I: Predicting the hourly total number of rented bikes in the capital bike sharing system

**Figure 6:**
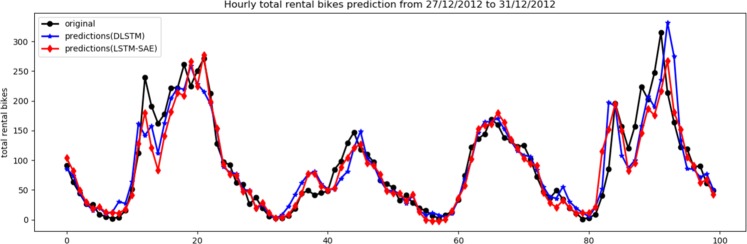
Original data vs. prediction for case 1 with 1 layer.

**Figure 7:**
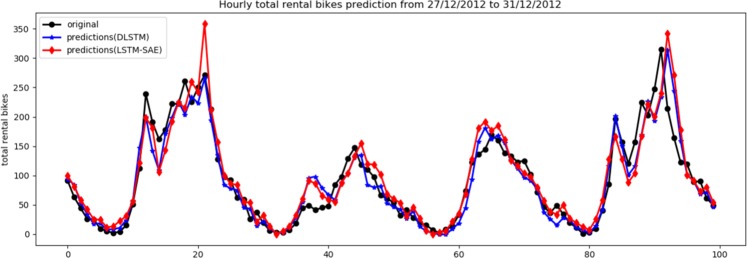
Original data vs. prediction for case 1 with 2 layer.

Table 1 shows the results of both models using one and two hidden layers. To avoid overfitting, we did not increase the number of hidden layers; in particular, we found that the error values did not change, and therefore, the performance is still the same. In addition, the table shows the best values of each parameter that show the best performance of each model. It is clear from Table 1 that the LSTM-SAE model outperforms the DLSTM model on the three performance measures. Given that both models have a deep structure using the original LSTM, these results assert the impact of adopting the approach of the unsupervised pre-trained LSTM instead of the randomized initialization for LSTM cells. For visual assessment, the relation between the original data and their prediction for the LSTM-based SAE model and the DLSTM model is illustrated in Figs. 6 and 7 for one hidden layer and two hidden layers, respectively. For clarity, these two figures show the last 100 observations of the testing data.

**Table 1:** The results of DLSTM^5^ and LSTM-SAE using dataset of case study-I.

Model	No. of hidden layer	No. of hidden units	Dropout	lag	batch	RMSE	MAE	SMAPE
DLSTM	1	^47^	0.4	20	146	52.062	32.468	**12.088**
DLSTM	2	^23,24^	0.3	25	219	**49.811**	**31.524**	12.183
LSTM-SAE	1	^33^	0.1	30	219	49.389	32.192	13.878
LSTM-SAE	2	^5,47^	0.1	30	73	**46.927**	**30.041**	**11.646**

### Case study-II: Predicting the hourly PM2.5 concentration in the air of Beijing

**Figure 8:**
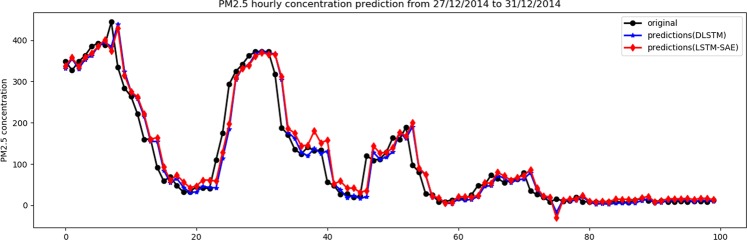
Original data vs. prediction for case 2 with 1 layer.

**Figure 9:**
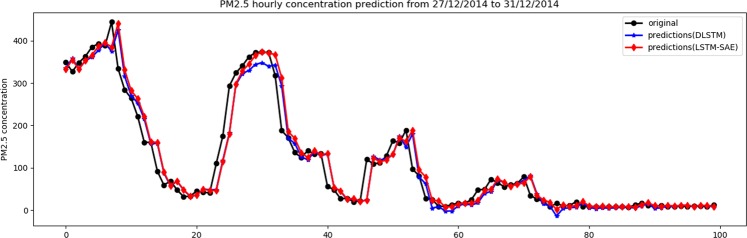
Original data vs. prediction for case 2 with 2 layer.

In a similar way, Table 2 shows the results of both models using one and two hidden layers. Again, it is obvious that the LSTM-SAE model outperforms the DLSTM model on two performance measures, namely, *MAE* and *SMAPE*. Regarding the *RMSE* measure, we found that the DLSTM outperforms the LSTM-SAE, where the former shows 23.75 and the latter shows 24.04, i.e., a minor difference. The visual relation between the original data and their predictions using both models is illustrated in Figs. 8 and 9 for one hidden layer and two hidden layers, respectively, for only the last 100 observations of testing data.

**Table 2:** The results of DLSTM^5^ and LSTM-SAE using data set of case study 2.

Model	No. of hidden layer	No. of hidden units	Dropout	lag	batch	RMSE	MAE	SMAPE
DLSTM	1	^47^	0.2	30	60	23.993	**12.124**	**10.919**
DLSTM	2	^3,42^	0.2	30	73	**23.750**	12.452	12.181
LSTM-SAE	1	^20^	0.1	30	219	**23.907**	12.509	11.052
LSTM-SAE	2	^25,26^	0.3	25	146	24.041	**12.060**	**9.864**

## Results Analysis and Discussion

In the previous section, we validated the performance of the proposed LSTM-SAE model against our previous model DLSTM^5^ to solve the MTS forecasting problem. In this section, we will validate both models against state-of-the-art machine learning models that are used to solve the same problems using the same datasets. We already overviewed these reference models in the section of Related Work. All validations are based on the three performance metrics *RMSE*, *MAE*, and *SMAPE*. As we explained in the performance measures subsection, the performance metric *MAE* is more accurate than *RMSE*, and *SMAPE* is more accurate and efficient than both of them. The reason for this is widely demonstrated in the literature, indicating that the percentage error measures are the most appropriate tools to assess the performance of forecasting models^60^. Accordingly, in this discussion section, we will mainly rely on the values of the *SMAPE* measure if its values are available. However, if *SMAPE*’s value is not available in the corresponding reference, we will rely first on the *MAE* values, and then we will rely on the *RMSE* values. Additionally, since both case studies have similar experimental conditions, we will base our analysis on the error values or performance comparison across both cases.

### LSTM-SAE vs. DLSTM

For easier comparison, we will merge both Tables 1 and 2 for both case studies into one table. It is clear from Table 3 that the proposed model LSTM-SAE outperforms our previous model DLSTM^5^ in all performance measures except in the *RMSE* measure in case study-II. On the scale of this measure, the DLSTM gains 23.7, whereas the LSTM-SAE gains 24.1, whilst in the other two measures, *MAE* and *SMAPE*, the latter approach outperforms the former. If we adhere to our justifications about the three performance measures, then we can confirm the superiority of the LSTM-SAE over the DLSTM in solving MTS forecasting problems.

**Table 3:** The comparison between DLSTM^5^ and LSTM-SAE in case of 2 layers in both case studies.

Case Study	DLSTM			LSTM-SAE		
	RMSE	MAE	SMAPE	RMSE	MAE	SMAPE
I	49.811	31.524	12.183	**46.927**	**30.041**	**11.646**
II	**23.750**	12.452	12.181	24.041	**12.060**	**9.864**

These results clearly show the impact of stacking LSTMs in an autoencoder fashion as a kind of pre-trained DLSTM, and its performance is better than the original DLSTM that depended on randomized initialization to the units of the LSTM. In other words, the unsupervised pre-training approach enhances the DLSTM’s performance better than the supervised approach of the DLSTM^5^. The reason is because UTS problems usually include one variable or one feature, where there is no correlation with other variables. Therefore, it is expected that the DLSTM will perform well in such regression problems. However, there are many variables in MTS regression problems and they are highly correlated as well. In such complex situations, the random initialization of cells will lead the learning algorithm to become trapped in different local minima, depending on the values of the other parameters’ initialization. This coincides with what is demonstrated in the machine learning literature, indicating that training deep architectures with several layers using random weights initialization and supervised training provides worse results than training shallow architectures^18,19^.

Furthermore, we noticed that the unsupervised pre-training approach assists the model to converge faster than the supervised approach of DLSTM. Table 4 lists the number of epochs required from each model to converge in both case studies; in the case of the LSTM-SAE model, this is the number of epochs in the fine-tuning phase. We extended the list to show number of epochs required up to three hidden layers to track the convergence closely. In case study-I, the DLSTM model needs **500** epochs to converge, whereas LSTM needs **355** epochs to converge. Likewise, in case study-II, the DLSTM model needs **1182** epoch, whereas the LSTM-SAE needs **197** epochs to converge. Of course, these parameters are mainly dependent on the dataset, but they provide us with an indication about computational requirements for each model.

**Table 4:** The number of epochs needed for the two models to converge in both cases studies.

Case Study	No. of hidden layers	DLSTM	LSTM-SAE
		No. of epochs	No. of epochs
I	1	299	**285**
	2	414	**255**
	3	500	**355**
II	1	**123**	244
	2	**127**	463
	3	1182	**197**

Overall, the unsupervised pre-training indeed enhances the performance of the DLSTM model and leads to better convergence but with the cost of increasing the overall training of the LSTM-SAE model because of the two phases of training (pre-training and fine-tuning), and this is the trade-off with the improved performance.

### LSTM-SAE vs. reference models

Towards an overall evaluation and analysis for the proposed model, Table 5 shows a comparison for the proposed model with other reference models using the same dataset of case study-I to solve the same problem^43^. In principal, a comparison with the results of other models in different experimental conditions is common in the machine learning community, as long as the comparison is based on tracking the computational performance of each model to solve the same problem using the same dataset across the same performance measures. The benefit of such a comparison is that it will confirm the validation of the proposed model against several counterparts. As the authors of reference models^43^ used the *RMSE* measure, similar to our paper here, we will compare their results with ours for the *RMSE* measure only, as shown in Table 5.

**Table 5:** Comparison of the proposed model with other models using data set of case study-I.

Baseline Model	RMSE	MAE	SMAPE
LSTM-SAE **(Proposed)**	**46.92**	**30.04**	**11.64**
DLSTM^5^	49.81	31.52	12.18
AIS-RT (Presented in)^43^	62.98	—	—
Reg Tree^43^	69.20	—	—
Ensemble Regression Tree^43^	82.59	—	—
Ridge Regression^43^	79.32	—	—
Adaboost Regression^43^	102.46	—	—
Gradient Boosting Regression^43^	312.69	—	—
Random Forest Regression^43^	336.17	—	—

The reference models assessed here are: artificial immune system with regression trees (AIS-RT), regression tree (Reg Tree), ensemble regression tree, ridge regression, adaboost regression, gradient boosting regression, and random forest regression. It is clear that the proposed LSTM-SAE model, and even our previous DLSTM model, outperform the other reference model^43^, which is AIS-Tree, as well as the other reference models developed and compared in the same article^43^. Therefore, we can conclude that the proposed LSTM-SAE model provides a greater performance improvement than ensemble approaches on regression trees.

In case study-II, we assess our two models against several recently reported approaches used to solve the same problem using the same dataset. Table 6 shows a comparison with the results of other reference models reported in the literature. We conduct the comparison using the performance measures that are used in these reference papers and the current paper as well, namely, *RMSE*, *MAE*, and *SMAPE*. Again, according to each measure of accuracy and efficiency reported in the literature^58,59^, we will rely first on the values of *SMAPE*, then on the values of *MAE*, then finally on the values of *RMSE*.

**Table 6:** Comparison of the proposed model with other models using data set of case study-II.

Model	RMSE	MAE	SMAPE
LSTM-SAE **(Proposed)**	24.04	**12.06**	**9.86**
DLSTM^5^	23.75	12.45	12.18
GC-LSTM^45^	**22.41**	13.72	—
LSTM^45^	27.19	16.82	—
FNN^45^	28.8	17.77	—
MLR^45^	38.03	24.35	—
Wavelet-ARIMA^46^	26.43	19.82	—
Wavelet-ANN^46^	32.87	24.20	—
CNN-LSTM^44^	24.23	14.74	—
CNN^44^	24.60	16.13	—
CE-WOA-LSSVM^47^	—	—	11.34
GRNN^47^	—	—	22.77
ARIMA^47^	—	—	39.44

The reference models assessed here are: graph convolutional network-LSTM (GC-LSTM), the original LSTM, the original feed forward neural networks (FNN), the original multiple linear regression (MLR), wavelet auto-regressive integrated moving average (wavelet-ARIMA), wavelet-artificial neural networks (wavelet-ANN), CNN-LSTM, the original CNN, complete ensemble-least squares support vector machine (LSSVM), the original general regression neural network (GRNN), and the traditional and original auto-regressive integrated moving average (ARIMA). These models are reported originally in many articles in the literature.

It is clear from Table 6 that the proposed LSTM-SAE model and our previous model DLSTM outperform all other models on the level of both *MAE* and *SMAPE* performance measures, which confirms the superiority of our model (the models in Zhao *et al*.^47^ is measured on *MAPE* that is the baseline measure and belong to same category of the *SMAPE* measure that is used in Table 6). There is only one exception, where the GC-LSTM^45^ model outperforms our models and all other reference models on the scale of the RMSE performance measure. Again, we can conclude that the LSTM-SAE model provides a greater performance improvement than several machine learning baseline and top-of-the-line models.

## Conclusion

In this paper, we proposed a pre-trained LSTM-based stacked autoencoder (LSTM-SAE) approach in a layer-wise unsupervised learning fashion to replace the random weight initialization approach of the units of the deep LSTM (DSLTM) model that we previously presented. The proposed approach is tested and validated to solve the multivariate time series forecasting problem using different two case studies and different datasets. The experimental results clearly show that the layer-wise pre-training approach improved the performance of DLSTM and led to better and faster convergence. In addition, the layer-wise pre-training fashion is more suitable to problems that include multiple and correlated variables. It could learn the latent features automatically from the dynamically changing multivariate inputs that are included in the two case studies treated in the paper. This performance is further confirmed by its favourable comparative performance with several machine learning baseline models that solve the same case studies. However, the side effect of the unsupervised pre-training approach lies in the longer training time that is required to sequentially implement the two phases: the pre-training phase and the fine-tuning phase. This will motivate us to combine a suitable selective attention mechanism to improve the training time and reduce the time complexity.
